# Untargeted Metabolomics for Metabolic Diagnostic Screening with Automated Data Interpretation Using a Knowledge-Based Algorithm

**DOI:** 10.3390/ijms21030979

**Published:** 2020-02-01

**Authors:** Hanneke A. Haijes, Maria van der Ham, Hubertus C.M.T. Prinsen, Melissa H. Broeks, Peter M. van Hasselt, Monique G.M. de Sain-van der Velden, Nanda M. Verhoeven-Duif, Judith J.M. Jans

**Affiliations:** 1 Section Metabolic Diagnostics, Department of Genetics, University Medical Centre Utrecht, Utrecht University, Lundlaan 6, 3584 EA Utrecht, The Netherlands; 2 Section Metabolic Diagnostics, Department of Child Health, Wilhelmina Children’s Hospital, University Medical Centre Utrecht, Utrecht University, Lundlaan 6, 3584 EA Utrecht, The Netherlands

**Keywords:** untargeted metabolomics, inborn errors of metabolism, IEM, direct-infusion high-resolution mass spectrometry, automated data interpretation, next generation metabolic screening, diagnostics

## Abstract

Untargeted metabolomics may become a standard approach to address diagnostic requests, but, at present, data interpretation is very labor-intensive. To facilitate its implementation in metabolic diagnostic screening, we developed a method for automated data interpretation that preselects the most likely inborn errors of metabolism (IEM). The input parameters of the knowledge-based algorithm were (1) weight scores assigned to 268 unique metabolites for 119 different IEM based on literature and expert opinion, and (2) metabolite Z-scores and ranks based on direct-infusion high resolution mass spectrometry. The output was a ranked list of differential diagnoses (DD) per sample. The algorithm was first optimized using a training set of 110 dried blood spots (DBS) comprising 23 different IEM and 86 plasma samples comprising 21 different IEM. Further optimization was performed using a set of 96 DBS consisting of 53 different IEM. The diagnostic value was validated in a set of 115 plasma samples, which included 58 different IEM and resulted in the correct diagnosis being included in the DD of 72% of the samples, comprising 44 different IEM. The median length of the DD was 10 IEM, and the correct diagnosis ranked first in 37% of the samples. Here, we demonstrate the accuracy of the diagnostic algorithm in preselecting the most likely IEM, based on the untargeted metabolomics of a single sample. We show, as a proof of principle, that automated data interpretation has the potential to facilitate the implementation of untargeted metabolomics for metabolic diagnostic screening, and we provide suggestions for further optimization of the algorithm to improve diagnostic accuracy.

## 1. Introduction

Untargeted metabolomics is readily finding its way into the metabolic diagnostic laboratory. In the near future, next generation metabolic screening (NGMS) through untargeted metabolomics may become the preferred approach to address diagnostic requests for patients referred to the metabolic diagnostic laboratory for basic metabolic screening, as advocated by the studies that demonstrated the diagnostic value of untargeted metabolomics for NGMS [1,2,3,4]. However, data interpretation of untargeted metabolomics analyses is labor-intensive due to the extensive amounts of data that are generated, the non-quantitative nature of the data, the lack of metabolite reference ranges, and the large number of metabolite alterations, of which the diagnostic significance remains to be elucidated [1,2,3,4]. This prevents metabolic laboratories from implementing a potentially powerful technique for NGMS. 

In addition, most untargeted metabolomics analyses are developed to detect hydrophilic, small-molecule metabolites, in either plasma [1,2,3], dried blood spots (DBS) [3], or cerebrospinal fluid (CSF) [4]. Therefore, these methods are currently only capable of correctly identifying inborn errors of metabolism (IEM) with hydrophilic small-molecule biomarkers in blood or CSF [1,2,3,4]. Many IEM, such as several lysosomal and peroxisomal disorders, lack these types of biomarkers, meaning that these IEM cannot be detected (yet) using these methods. For this reason, it is not yet possible to exclude IEM based on current NGMS methods. 

To facilitate further the implementation of untargeted metabolomics as a NGMS method, we developed an automated data interpretation approach that preselects the most likely IEM by designing a knowledge-based algorithm. As a proof of principle, we here demonstrate its diagnostic value. 

## 2. Results

### 2.1. Training Sets, Optimization Sets and Patient Samples

Automated data interpretation resulted in the correct diagnosis being present in the differential diagnoses (DD) in 78% of the 110 DBS samples in the training set and in 79% of the 86 plasma samples in the training set (Table 1 and Table S1). The correct diagnosis ranked first in 42% (DBS) and 33% (plasma) of the samples, and the median lengths of the DD were 8 (DBS) and 12 (plasma) IEM (Table 1 and Table S1). In the DBS training set, the correct diagnosis was absent in the DD in 24 samples of 12 patients harboring 9 IEM (Table S1). For each sample, the reason for not correctly preselecting the diagnosis is explained in Table S1. In 14/24 samples, this was due to the sample selection: the sample was drawn when the patient was under treatment and the diagnostic biochemical alterations had normalized. In 5/24 samples this was as a result of the detection method: Z-scores were in the normal range while targeted analyses demonstrated the presence of—often subtle—diagnostic biochemical alterations. In another 5/24 samples, this was caused by a combination of sample selection and the method: targeted analyses showed only mild biochemical alterations, resulting in normal Z-scores (Table S1). In the plasma training set, the correct diagnosis was not in the DD in 18 samples of 11 patients harboring 8 IEM (Table S1). In line with the missed diagnoses in DBS, this was either due to the sample selection (7/18), the method (8/18), or a combination thereof (3/18) (reasons listed in Table S1).

In the DBS optimization set, automated data interpretation resulted in the correct diagnosis in the DD in 71% of the 96 samples (Table 1), including 38 different IEM, of which 19 were not included in the DBS training set (Supplementary Table S1). The correct diagnosis ranked first in 40%, and the median length of the DD was 8 IEM (Table 1 and Table S1). The correct diagnosis was not in the DD of the 28 samples of 28 patients harboring 20 IEM, and reasons for not correctly preselecting these diagnoses are listed in Table S1. These reasons included the diagnostic algorithm (1/28), a combination of the algorithm and the method (4/28), the method (14/28), and a combination of sample selection and the method (2/28). For 7/28 samples, the reason for missing the correct diagnosis was not known, as the diagnostic metabolites that the algorithm is based on were not quantified using targeted analyses (Table S1).

### 2.2. Validation Set, Patient Samples

In the plasma validation set, automated data interpretation included the correct diagnosis in the DD in 72% (Table 1), comprising 44 different IEM, of which 24 IEM were not included in the plasma training set. The correct diagnosis ranked first in 37% of the samples and in the top 3 in 57% of the samples, and the median length of the DD was 10 IEM (Table 1). In 32 samples of 32 patients harboring 21 IEM, the diagnosis was not in the DD (Table S1). This was either due to the diagnostic algorithm (10/32), to a combination of the method and the algorithm (3/32), to the method (12/32), or to the sample selection (1/32). For 6/32 samples, the reason for missing the correct diagnosis could not be determined, as the diagnostic metabolites that the algorithm is based on were not quantified using targeted analyses (all reasons have been explained in Table S1). Proposed further changes to the expected library based on the results of the validation set, as listed in Table S1, could improve the diagnostic yield of the algorithm to 77%, as they would result in the correct diagnosis in the DD in another 6 samples, comprising 4 IEM (demonstrated in Table S1, Patient validation set plasma). 

### 2.3. Performance Assessment of All Patient Samples

Automated data interpretation included the correct diagnosis in the DD in 75% (*n* = 305/407 of all included patient samples), comprising 53 different IEM. The correct diagnosis ranked first in 26% (*n* = 155/407) and in the top 3 in 59% (*n* = 240/407) of the patient samples. The categories storage disorders and disorders of peroxisomes and oxalate had a true positive rate of 100%, but both these categories only included two samples (Table S1). The diagnostic algorithm performed best among disorders of nitrogen-containing compounds and among disorders of lipids (80.3% and 79.8% true positive samples, respectively). The algorithm performed worse among the other common conditions and interferences, mitochondrial disorders of energy metabolism, and among disorders of carbohydrates (Table S1). 

### 2.4. Control Samples

For control samples, the median length of the DD was 2 IEM for DBS in both the training and optimization set, and 3 IEM for plasma in both the training and validation set (Table 2). In control DBS samples, IEM that were frequently noted in the DD of the training and optimization sets included aromatic L-amino acid decarboxylase deficiency (*n* = 17/171), Hartnup disorder (*n* = 12/171), and the condition “fasting” (*n* = 11/171) (Table S1). In control plasma samples, IEM that frequently arose in the DD of the training and validation sets included Hartnup disorder (*n* = 23/167), 3-phosphoserine phosphatase deficiency (*n* = 20/167), and the condition “fasting” (*n* = 20/167) (Table S1). None of the individuals whose samples were selected as control samples experienced any clinical problems that could, in retrospect, be related to any of the IEM included in the DD of these patients.

### 2.5. R Shiny App to Aid Insight in Automated Data Interpretation

The accessibility of the R Shiny app that was developed is demonstrated in Supplementary Video S1. This video shows how the app provides an interface to select patient samples and IEM in the DD, in order to see how the data was valued, which data was valued, and how the DD was created in the automated data interpretation (Supplementary Video S1). 

## 3. Discussion

New all-encompassing technologies, such as untargeted metabolomics, are finding their way to clinical applications. Interpretation of the extensive data sets, however, is laborious and should preferably be automated. Here, we provide a proof of principle, demonstrating that automated data interpretation using a knowledge-based algorithm has added value in the diagnostic use of untargeted metabolomics. The algorithm that we developed correctly reduces a list of 119 IEM and 7 other conditions and interferences to a DD, which consists of approximately 10 IEM, in 72% of the plasma samples. In 37% of the samples, the correct diagnosis ranked first and, in 57% of the samples, the correct diagnosis ranked in the top 3. As expected, the diagnostic algorithm performs best among disorders of nitrogen-containing compounds and disorders of lipids, achieving a true positive rate of approximately 80%. Moreover, we showed that we were able to correctly preselect 44 different IEM very rapidly and based on only one sample. Interestingly, in the DBS optimization set and in the plasma validation set, respectively, 19 and 24 IEM were correctly preselected, although these IEM were not included in the training set. This demonstrates that even without prior testing, IEM can be correctly preselected by the diagnostic algorithm, solely based on literature and expert knowledge on diagnostic metabolite alterations. 

We envision that the interpretation of the preselection that the diagnostic algorithm provides, should be performed by trained laboratory specialists, in line with the current workflow for results of targeted diagnostic platforms. Provided that these laboratory specialists are specialized in IEM, they can appraise the significance of the observed biochemical alterations and relate them to the patient’s phenotype in order to judge for each IEM in the DD whether the clinical phenotype potentially fits the diseases and whether this IEM should indeed be considered as a diagnosis in the patient. Based on this judgement, laboratory specialists could recommend second-tier tests to confirm or refute IEM in the DD.

Many IEM share the same biomarker or set of biomarkers critical for the diagnosis. However, as the specific defect varies between these IEM, other metabolites have the potential to corroborate a certain diagnosis, although these metabolites are in general not able to confirm or refute a diagnosis with absolute certainty. This is exemplified by disorders of the phenylalanine and tetrahydrobiopterin metabolism. We included three IEM, all characterized by an increase of phenylalanine: phenylketonuria, dihydropteridine reductase deficiency, and DNAJC12 deficiency (Table S1). All disorders of the phenylalanine and tetrahydrobiopterin metabolism are present in the DD of the included samples, as none of the additional metabolites are able to confirm or refute a diagnosis with certainty. However, these metabolites can provide supportive evidence to corroborate a diagnosis. In the DD of all these samples, phenylketonuria ranks first, as the weight of phenylalanine in this IEM is the highest (Table S2). However, a decrease of homovanillic acid (Z-score 2.95, rank 3) in P038.1 with dihydropteridine reductase deficiency and a decrease of 5-hydroxyindoleacetic acid (Z-score -1.64, rank 24) in P070.1 with DNAJC12 deficiency ensure that, in both these samples, DNAJC12 deficiency ranks second and dihydropteridine reductase deficiency ranks third. In contrast, in samples of patients with phenylketonuria, these metabolites were normal and DNAJC12 deficiency and dihydropteridine reductase deficiency can also rank fourth or fifth. This example demonstrates that, next to the main biomarker(s) for a disease, additional biomarkers—captured by untargeted metabolomics and considered by the diagnostic algorithm—can support the likeliness of a certain diagnosis.

For a first-tier test for patients referred for basic metabolic screening, a diagnostic value of ~70% of the preselection algorithm for untargeted metabolomics is still insufficient. However, routine targeted basic metabolic diagnostic screening, the current gold standard for metabolic diagnostics, is not perfect either, as IEM diagnoses can be overlooked when only assessing a selection of biomarkers in a single blood sample. This imperfection of the current gold standard is corroborated by the annual report of 2017 of the European Research Network for the evaluation and improvement of the screening, diagnosis, and treatment of Inherited Disorders of Metabolism, which reports that 82.9% of participating laboratories obtained satisfactory performance in all of the external quality assessment schemes they participated in [5]. False-negative findings of the diagnostic algorithm, i.e.; IEM that were incorrectly left out of the IEM preselection in the DBS optimization set and the plasma validation set, were, for example, galactosaemia type I and methyltetrahydrofolate reductase deficiency (Table S1). For galactosaemia type I, this is most likely due to the fact that, in direct infusion, an observed m/z can account for multiple metabolite annotations since metabolites can have multiple isomers. In general, this does not result in any data interpretation problems or incorrect diagnoses, since most m/z are dominated by a single metabolite of significant abundance [3]. However, the m/z of galactose and galactose-1-phosphate are most likely dominated by glucose and glucose-1-phosphate, thereby masking the increases of galactose and galactose-1-phosphate. The reason for not including methyltetrahydrofolate reductase deficiency in the DD is that a methanol-based sample extraction only captures the unbound fraction of homocysteine, which is not an accurate representation of the total homocysteine concentration in blood [3]. 

Conversely, some IEM in the DD of control samples were more common than others (Table S1). These IEM could be considered false-positive findings. However, slight alterations of amino acids, organic acids, and acylcarnitines that do not correspond to any specific IEM diagnosis are also frequently detected using routine targeted basic metabolic diagnostic screening, but these insignificant findings in general do not cause any diagnostic dilemmas. The diagnostic algorithm frequently included aromatic L-amino acid decarboxylase deficiency. The presence of this IEM in the DD might be an artefact caused by the use of L-Dopa, which is frequently used in pediatric patients for its inotropic function. We noted this potential artefact in the “artefact disclaimer” (Table S2). Likewise, for Hartnup disorder, we noted in the artefact disclaimer that this disease is caused due to a defective urinary transporter and that this disease is hard, although not impossible (Table S1, Plasma validation set, Patient P054.1), to detect in blood (Table S2). Additionally, the diagnosis of Hartnup disorder, as well as those of fasting and serine synthesis defects are mainly based on amino acid decreases. The frequent detection of amino acid decreases is explained by the fact that many samples were drawn when patients were in a fasting state, and, like in targeted diagnostics, this is not expected to cause any diagnostic dilemmas.

A limitation of this study comprises the sample selection. The algorithm was designed using both DBS and plasma samples that were also used for the development of our method [3]. With the aim to validate the designed algorithm, DBS and plasma samples were collected for the validation set. However, during the analysis of the DBS validation samples, it was noted that more optimization could be done that would improve the algorithm to a significant extent. It was then decided that the DBS set would serve as an optimization set, and that the plasma sample set would serve as a final validation set. Ideally, one would collect a new DBS validation set, but due to the rarity of IEM and considering the preference for samples drawn when patients were not under treatment, this was not feasible. In addition, although a sample size of 407 IEM samples is impressive, a sample size of 3 samples per IEM is rather small. For more common IEM, more samples were available, but it was decided to not include these samples as inclusion would skew the robustness of the model since these IEM would influence the model more than rarer IEM. Lastly, despite a sample size of 407 IEM samples, we were not able to validate the preselection accuracy of the diagnostic algorithm for every IEM included in the IEM panel since we did not have samples of all IEM. 

Based on a thorough analysis of the successes and failures of the diagnostic algorithm, we propose six ways to further improve the robustness of the diagnostic algorithm. First, we advocate (1) expanded testing of the algorithm, including samples of IEM that were not tested yet, and subsequent further optimization of the algorithm, including the proposed changes in Table S1 (Table S1, Patient validation set plasma). The potential value of expanded testing is exemplified by carbamoylphosphate synthase I deficiency. This IEM was not incorporated in the training or optimization sets, but only in the validation set. In the expected library, an increase of L-glutamine was considered indispensable for the diagnosis. However, this resulted in missing this IEM in the preselection, while changing the importance of L-glutamine from critical to important influence resulted in the correct inclusion of this diagnosis in the DD (Table S1, Patient validation set plasma). We showed that the proposed further changes to the expected library based on the results of the validation set, could theoretically improve the diagnostic yield of the algorithm to 77% (Table S1, Patient validation set plasma), corroborating that the algorithm can be further optimized by expanded testing of additional IEM samples. Second, to reduce the number of false-negative findings, we suggest (2) investigating whether a metabolite-specific Z-score cut-off value for increases and decreases could lead to a reduction of the DD provided by the algorithm. For most diagnostic metabolites a Z-score cut-off value higher than 1.6, or lower than –1.2, would increase the specificity of the algorithm. However, the accurate implementation of this improvement requires expanded testing of the range of metabolite increases per IEM, for common IEM as well as for rarer IEM, requiring a large sample size per IEM. Third, (3) the addition of phenotypic data to the algorithm could theoretically lead to a reduction of the DD provided by the algorithm. Computational phenotype analysis has been proven successful in exome prioritization [6,7] and is likely to also have great potential for the prioritization of IEM, provided that a patient’s phenotype is described as comprehensively as possible. Fourth, we support the (4) addition of biomarkers that are identified by untargeted metabolomics studies to the library [3,8,9,10,11], (5) the inclusion of additional [12] or newly discovered IEM, and (6) the testing of the algorithm in other body fluids, like cerebrospinal fluid [4] or urine [13]. 

An important strength of our study is that the diagnostic algorithm is based on an IEM panel. In line with gene panels in genetic screening approaches, this could facilitate a conclusion such as “NGMS did not reveal any indication of an IEM included in the IEM panel” provided that the diagnostic value is sufficiently high. A second strength of the diagnostic algorithm is its independence of the type of untargeted metabolomics method that is used. Any metabolic diagnostic laboratory can test, use, and improve the diagnostic algorithm as long as metabolite identification can be converted to HMDB codes [14] and metabolite intensities can be converted to Z-scores. Moreover, the development of the R Shiny application ensures that we can present the algorithm as an easily accessible diagnostic tool for NGMS. In addition, since the number of known IEM is, in conjunction the number of potential biomarkers for IEM [3,8,9,10,11], expanding at an unprecedented pace [12], it gets increasingly hard for laboratory specialists to keep knowledge up-to-date. The expected library, which serves as the input for the diagnostic algorithm, can be easily expanded with newly discovered IEM, as well as new biomarkers, provided that the markers included in the library are validated and that the algorithm can correctly preselect the IEM in a patient sample. 

## 4. Materials and Methods 

### 4.1. Development of an IEM-Panel and Automated Data Interpretation

**Figure 1:**
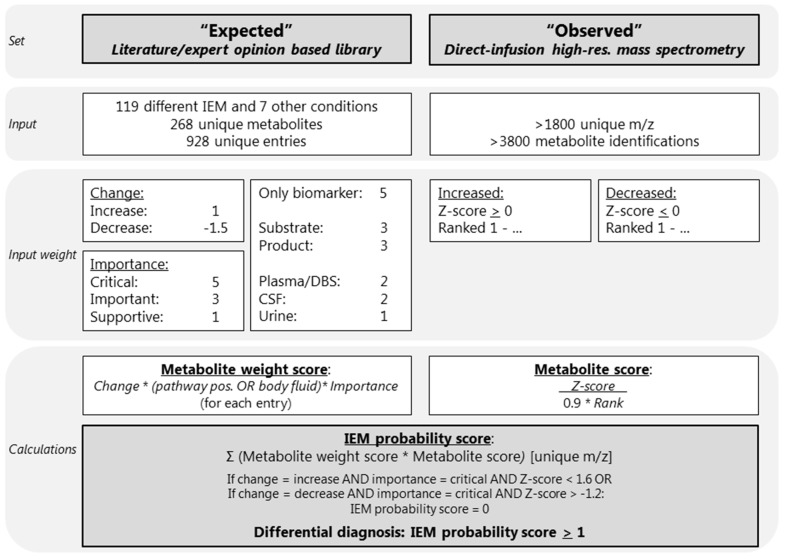
Overview of the diagnostic algorithm. CSF: cerebrospinal fluid; DBS: dried blood spots; IEM: inborn error of metabolism; m/z: mass to charge ratio. The * indicates multiplication. The Rank (Ranked 1-…) is calculated as followed: positive *Z*-scores were ranked from the maximum *Z*-score to zero (the highest *Z*-score observed ranked at position 1), and negative *Z*-scores were ranked from the minimum *Z*-score (ranking at 1) to zero.

We developed a diagnostic knowledge-based algorithm. The first input parameter for this algorithm was an “expected library” consisting of current knowledge about metabolite alterations for the IEM included in IEM panel. The second input parameter, here called “observed”, consisted of the results of the untargeted metabolomics method. The output of the algorithm was a ranked list of differential diagnoses (DD), per patient sample. An overview of the algorithm is visualized in Figure 1.

**Figure 2:**
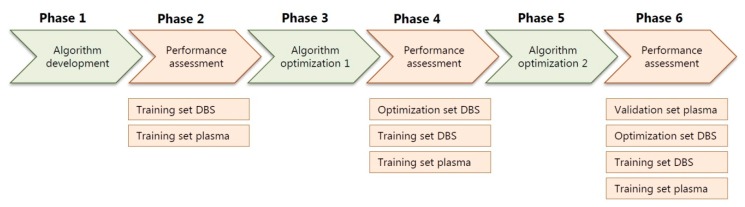
Flowchart of the six phases of the development and optimization of the diagnostic algorithm, with in-between assessments of the performance of the algorithm in the training sets, DBS optimization set and plasma validation set. DBS: dried blood spots.

The algorithm, including the expected library, was developed in six phases (Figure 2). First, a preliminary version of the algorithm was developed, followed by testing of the diagnostic value using a training set containing both DBS and plasma samples. Next, the algorithm was further optimized, again, followed by testing of the diagnostic value, now using an optimization set of DBS samples. Finally, the algorithm was again optimized, followed by a final assessment of the preselection accuracy using a validation set consisting of plasma samples (Figure 2). All presented results were determined using the final version of the algorithm (Figure 2).

Flowchart of the six phases of the development and optimization of the diagnostic algorithm, with in-between assessments of the performance of the algorithm in the training sets, DBS optimization set and plasma validation set.

### 4.2. Patient Inclusion

The IEM that were of interest to this study were IEM with one or more known small-molecule biomarkers in blood (Table S2). This mainly included disorders of nitrogen-containing compounds, disorders of vitamins, cofactors, metals and minerals, disorders of carbohydrates, and disorders of lipids according to the nosology of Ferreira et al. [12] (Table S2). Patients affected with these IEM were included when at least one remnant DBS or plasma sample was available in the metabolic diagnostic laboratory of the University Medical Centre Utrecht. All patients, or their legal guardians, approved the possible use of their remaining samples for method validation, in agreement with institutional and national legislation. All followed procedures were in accordance with the ethical standards of the University Medical Centre Utrecht and with the Helsinki Declaration of 1975, as revised in 2000. 

### 4.3. Sample Inclusion

Up to three samples per patient per IEM were included in each sample set. The training set consisted of the samples that were used for the development of our previously published untargeted metabolomics method [3]. This set included 110 DBS of 42 patients harboring 23 different IEM and 86 plasma samples of 38 patients harboring 21 different IEM. In addition, this set included 105 DBS of 30 individuals and 84 plasma samples of 28 individuals that served as control samples, which are defined as samples from individuals in whom an IEM was excluded after a thorough routine diagnostic work-up [3]. All included samples are listed in Table S1 (Table S1). 

For the DBS optimization set and for the plasma validation set, one sample per patient per IEM was included. Preferably, samples of patients who did not receive relevant treatment at the time of sampling were selected. When these samples were not available, samples were selected in which diagnostic metabolite alterations for an IEM were measured by targeted diagnostics before inclusion. The DBS optimization set consisted of 96 DBS of 96 patients harboring 53 different IEM. This set also included 66 DBS of 48 individuals that served as control samples. None of the patient DBS samples included in the optimization set were included in the training set. The plasma validation set included 115 plasma samples of 115 patients harboring 58 different IEM, and 83 plasma samples of 28 individuals that served as control samples. None of the patient plasma samples included in the validation set were included in the training set. Control samples of the optimization and validation sets were identical to the control samples used in the training set. All included samples are listed in Table S1 (Table S1). 

### 4.4. Input Parameter: Expected Library

Based on extensive literature research, the Online Mendelian Inheritance in Man (OMIM), IEMbase version 1.4.3 (www.iembase.org), the database of the Metabolic and Genetic Information Centre (www.metagene.de), and the expert opinions of four trained laboratory specialists, a database was constructed containing the expected metabolite alterations of 268 unique metabolites assigned to 119 different IEM and 7 other common conditions and interferences, with a total of 928 entries (Figure 1, Table S2). These other conditions and interferences included liver failure, renal failure, fasting, haemolysis, prolonged plasma storage, dietary cobalamin deficiency, and folate deficiency (Table S2). For each of the 119 IEM we noted the following: disease category and disease group according to the nosology of Ferreira et al. [12]; disease, gene, and protein function; the OMIM number including a link; a link to the clinical phenotype from the Human Phenotype Ontology; the estimated incidence of the disease, which was retrieved from OMIM, Orphanet, and from literature research; and an artefact disclaimer that describes potential artefacts that might cause alterations of the diagnostic metabolites (Table S2). Each of the 928 metabolite entries contained the metabolite name, the Human Metabolome Database (HMDB) code [14] including a link, the mass to charge ratio (m/z), whether there were any endogenous isomers for the compound, the names of the endogenous isomers, and the expected metabolite alteration (increased or decreased) (Table S2). In addition, we also noted the following information per metabolite entry: in which body fluid the metabolite alteration is known to occur; whether the metabolite is either a substrate or a product of the affected enzyme; whether the metabolite is the only known biomarker; whether the metabolite is known to be increased or decreased; and the importance of the metabolite alteration (Table S2). 

A metabolite alteration in either plasma, DBS, or CSF was considered more important than a metabolite alteration in urine, as the algorithm was optimized and tested for plasma and DBS samples. Therefore, plasma, DBS, and CSF were assigned a value of 2 and urine was assigned a value of 1 (Figure 1, Table S2). To ensure that the most important metabolites are correctly valued, metabolites that were products or substrates of the affected enzymes were valued 3, and metabolites that were the only biomarker of the IEM were valued 5 (Figure 1, Table S2). To ensure that decreases, which are often more subtle than increases, were properly valued, an increase was valued 1 and a decrease was valued –1.5. Lastly, the importance of the metabolite alteration for the IEM diagnosis was noted in three categories: supportive was valued 1, important influence was valued 3, and critical was valued 5 (Figure 1, Table S2). Metabolite alterations considered to be critical for the diagnosis were labelled as indispensable for the diagnosis, indicating that when, for example, a decrease of serine is considered critical for the diagnosis of a serine deficiency, a serine deficiency cannot be in the DD if no decrease of serine is observed (Figure 1, Table S2). 

### 4.5. Input Parameter: Observed Metabolite Alterations Using Untargeted Metabolomics

Sample collection, sample preparation, and untargeted metabolomics analysis were performed as previously described [3]. In short, direct-infusion high resolution mass spectrometry was performed using a TriVersa NanoMate system (Advion, Ithaca, NY, USA) controlled by Chipsoft software (version 8.3.3, Advion), mounted onto the interface of a Q-Exactive high-resolution mass spectrometer (Thermo Scientific™, Bremen, Germany). For each sample, technical triplicates were analyzed, infusing each sample three times into the mass spectrometer. The scan range was 70 to 600 m/z in positive and negative mode. Mass peak annotation was performed by matching the m/z value of the mass peak with a range of two parts per million to the monoisotopic metabolite masses present in the HMDB, version 3.6 [14]. Annotations of metabolites that can occur endogenously were selected: >1800 unique m/z per batch, corresponding to >3800 metabolite identifications, in line with previous analyses [3,4,8]. For each mass peak per patient sample, the deviation from the intensities in the control samples was indicated by a Z-score, which was calculated by: Z-score = (intensity patient sample—mean intensity control samples)/standard deviation intensity control samples. Z-scores were calculated for both the patient and control samples. For each sample, positive Z-scores were ranked from the maximum Z-score to zero (the highest Z-score observed ranked at position 1), and negative Z-scores were ranked from the minimum Z-score (ranking at 1) to zero (Figure 1). 

### 4.6. Automated Data Interpretation

Automated data interpretation was performed as follows: for each metabolite entry in the expected library a “metabolite weight score” was calculated. This score was the product of the weights for an increase (1) or decrease (–1.5); only the biomarker (5), substrate, or product (3), the plasma, DBS, CSF (2), or urine (1); and the critical (5), important (3), or supportive (1) (Figure 1, Table S2). In addition, for all observed metabolite alterations measured by direct-infusion high-resolution mass spectrometry, a “metabolite score” was calculated as follows: Z-score/(rank × 0.9), as the rank, was considered slightly less important than the Z-score. Next, for each of the 119 IEM and 7 other conditions or interferences, an “IEM probability score” was calculated, summing the product of the metabolite weight score and the metabolite score for all metabolite entries with a unique m/z:

(1) $$IEM\;probability\;score=\underset{unique\;m/z}{\sum }\left(weight\;score\times \frac{Z-score}{rank*0.9}\right)$$

After the calculation of the IEM probability score, two scenarios were considered: (1) when a metabolite was expected to be increased and considered critical for the diagnosis, was not found increased (defined as Z-score < 1.6), the IEM probability score was set to zero, and, likewise, (2) when a metabolite was expected to be decreased and considered critical for the diagnosis, was not decreased (defined as Z-score > –1.2), and the IEM probability score was also set to zero (Figure 1).

All 119 IEM included in this algorithm, as well as the 7 common conditions and interferences, were classified based on the IEM probability score. An IEM was considered “very likely” when the IEM probability score was >50, “likely” when >10 and <50, “possible” when >1 and <10, “unlikely” when >0 and <1, and “very unlikely” when <0. The DD consisted of IEM classified as “very likely”, “likely”, or “possible”, corresponding to an IEM probability score of >1 (Figure 1). 

All arbitrary assigned values used in the diagnostic algorithm, including the metabolite weight scores, the consideration of the metabolite rank with factor 0.9, the Z-score cut-off values of < 1.6 and > −1.2, and the ranges for the probability scores, were defined based on trial and error in the algorithm development phase (Figure 2) by trying to achieve optimal results using both the DBS and plasma training sets.

### 4.7. R Shiny App to Aid Automated Data Interpretation

To aid the use of the diagnostic algorithm, an R Shiny application was developed. The expected library and the data file containing the untargeted metabolomics results were used as input files. The output of the application was an interface wherein a sample and an IEM in the DD can be selected. The calculations of the algorithm could be demonstrated for each sample and for each IEM in the DD. The R code for the R Shiny application is available in the Supplementary Information. The results of the untargeted metabolomics analysis of all IEM and control samples analyzed in the test, the optimization sets, and the validation sets are available in Tables S3–S8.

## 5. Conclusions

In conclusion, here, we demonstrate as a proof of principle that a diagnostic algorithm can correctly reduce a differential diagnosis of 119 IEM and seven other conditions and interferences to a median of 10 IEM in 72% of the plasma samples in the validation set. Moreover, we show that the algorithm can correctly preselect 44 different IEM very rapidly based on only a single sample. Although automated data interpretation is not yet flawless and still needs to be further optimized, we demonstrate that it could potentially facilitate the implementation of untargeted metabolomics for NGMS to a great extent.

## Figures and Tables

**Table 1:** Performance automated data interpretation for patient sample sets.

	Training Sets		Optimization Set	Validation Set
Matrix	DBS	Plasma	DBS	Plasma
Samples	110	86	96	115
Patients	42	38	96	115
IEM	23	21	53	58
Correct IEM in DD (*n*; %)	86/110; 78%	68/86; 79%	68/96; 71%	83/115; 72%
Correct IEM in top 3 of DD (*n*; %)	74/110; 67%	36/86; 42%	60/96; 63%	65/115; 57%
Correct IEM ranked first (*n*; %)	46/110; 42%	28/86; 33%	38/96; 40%	43/115; 37%
Length DD (median; (5th–95th))	8; [2–14]	12; [3–25]	8; [1–23]	10; [3–22]

DBS: dried blood spots; DD: differential diagnosis; IEM: inborn error of metabolism; *n*: total number; 5th: fifth percentile; 95th: ninety-fifth percentile.

**Table 2:** Performance automated data interpretation for control sample sets.

	Training Sets		Optimization Set	Validation Set
Matrix	DBS	Plasma	DBS	Plasma
Samples	105	84	66	83
Individuals	30	28	48	28
Length DD (median; (5th–95th))	2; (0–12)	3; (0–11)	2; (0–8)	3; (0–10)

DBS: dried blood spots; DD: differential diagnosis; 5th: fifth percentile; 95th: ninety-fifth percentile.

## Supplementary Materials

Supplementary materials can be found at https://www.mdpi.com/1422-0067/21/3/979/s1.

## Abbreviations

CSF	cerebrospinal fluid
DBS	dried blood spots
DD	differential diagnosis
HMDB	Human Metabolome Database
IEM	inborn error of metabolism
m/z	mass to charge ratio
NGMS	next generation metabolic screening
OMIM	Online Mendelian Inheritance in Man
